# Supplementary material for: Mechanisms governing the pioneering and redistribution capabilities of the non-classical pioneer PU.1
Source: Nat Commun. 2020 Jan 21;11:402. doi: 10.1038/s41467-019-13960-2 (PMC6972792; doi:10.1038/s41467-019-13960-2)
Supplement: Supplementary file 7 — Source data [file 41467_2019_13960_MOESM7_ESM.zip › Source_Data/Figure2/Figure2F_MetascapeResults/Enrichment_heatmap/HeatmapSelectedGO.pdf]

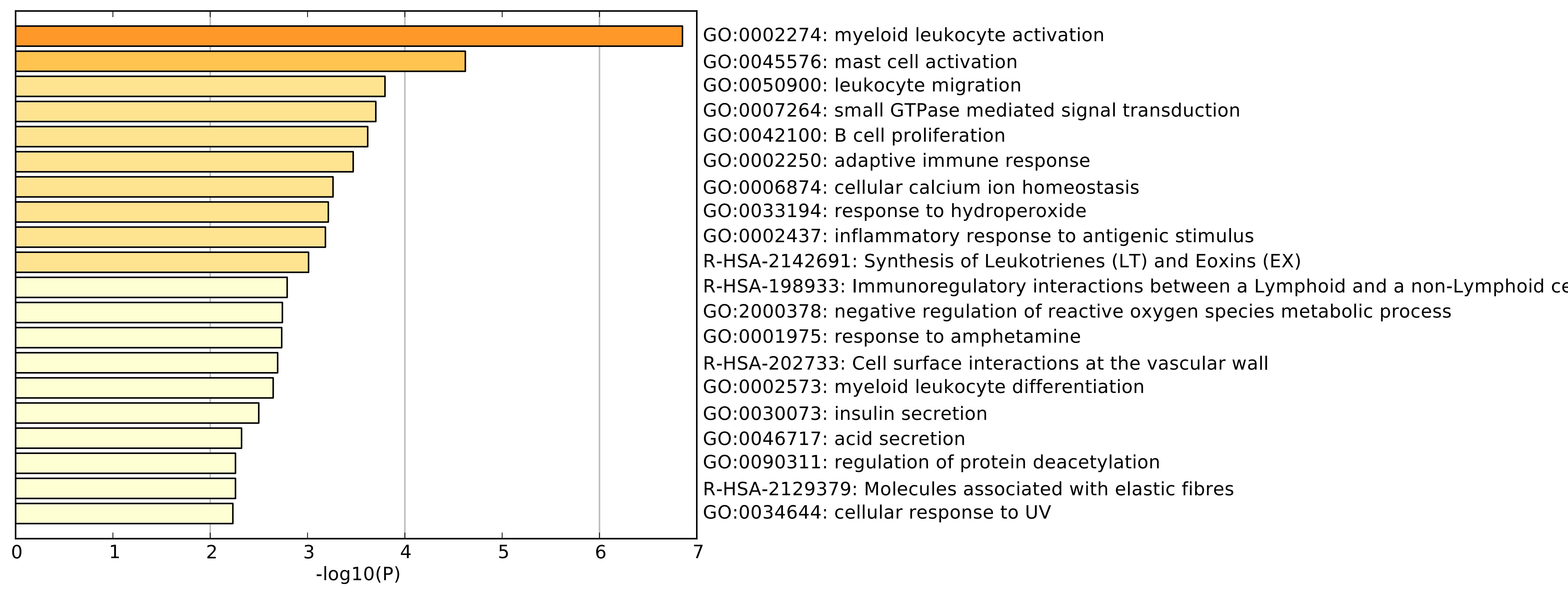

GO:0002274: myeloid leukocyte activation
GO:0045576: mast cell activation
GO:0050900: leukocyte migration
GO:0007264: small GTPase mediated signal transduction
GO:0042100: B cell proliferation
GO:0002250: adaptive immune response
GO:0006874: cellular calcium ion homeostasis
GO:0033194: response to hydroperoxide
GO:0002437: inflammatory response to antigenic stimulus
R-HSA-2142691: Synthesis of Leukotrienes (LT) and Eoxins (EX)
R-HSA-198933: Immunoregulatory interactions between a Lymphoid and a non-Lymphoid ce
GO:2000378: negative regulation of reactive oxygen species metabolic process
GO:0001975: response to amphetamine
R-HSA-202733: Cell surface interactions at the vascular wall
GO:0002573: myeloid leukocyte differentiation
GO:0030073: insulin secretion
GO:0046717: acid secretion
GO:0090311: regulation of protein deacetylation
R-HSA-2129379: Molecules associated with elastic fibres
GO:0034644: cellular response to UV
0
1
2
3
4
5
6
7
-log10(P)
